# Zoster Sine Herpete: two unusual cases of varicella-zoster reactivation with atypical complaints of acute chest pain and severe headache

**DOI:** 10.1186/s12879-023-08093-3

**Published:** 2023-04-18

**Authors:** Yilin Yang, Talha Mahmood, Afsheen H. Siddiqui, Muhammad A. Aziz

**Affiliations:** 1 Johns Hopkins University School of Medicine, Baltimore, MD USA; 2 Biological Sciences, Florida International University, Miami, FL USA; 3 Johns Hopkins Community Physicians, CIMS Hospitalist, Howard County General Hospital, Johns Hopkins Medicine, 5755 Cedar Lane, Columbia, MD 21044 USA

**Keywords:** Case report, Varicella-zoster virus, Zoster sine herpete, Herpes zoster, Meningitis

## Abstract

In this case report, we describe two unusual presentations of varicella-zoster virus (VZV) reactivation without rash, a condition known as Zoster Sine Herpete (ZSH). In Case 1, a 58-year-old woman presented with severe right-sided chest pain under her breast that radiated to the ipsilateral back. After the initial workup ruled out cardiac and musculoskeletal etiologies, the characteristic dermatomal distribution of pain made us suspect VZV reactivation. A diagnosis of ZSH was made with positive VZV IgG and IgM serologies and symptomatic relief after famciclovir treatment. In Case 2, a 43-year-old woman presented with a severe headache and resolved sharp right flank pain. She was diagnosed with varicella meningitis after cerebrospinal fluid showed positive VZV DNA. Intravenous acyclovir treatment resulted in symptom resolution. The most common presentation of VZV reactivation is Herpes Zoster, or shingles, making ZSH a frequently missed diagnosis. High clinical suspicion is warranted to prevent life-threatening complications of ZSH.

## Background

Zoster Sine Herpete (ZSH) is a consequence of varicella-zoster virus (VZV) reactivation, presenting with dermatomal neuralgia in the absence of rash [1]. More frequently, VZV reactivation results in Herpes Zoster (HZ), or shingles, characterized by vesicular eruption on an erythematous base and intense dermatomal pain [2, 3]. After chickenpox infection and/or vaccination, VZV can become latent in the dorsal root ganglia (DRG), cranial nerve ganglia, and enteric ganglia [4]. The latency is enabled by cell-mediated immunity. Factors such as advanced age, immunocompromising disease or medications, and psychological stress can therefore lead to loss of normal immune surveillance and consequent viral renaissance.

Since HZ is a common disease, ZSH, which shares the same pathophysiology, warrants attention in the differential diagnosis for dermatomal neuralgia. A thorough work up is needed to rule out other diagnosis causing similar symptoms and could also present with a rash like herpes simplex virus, impetigo, candidiasis, autoimmune disease, contact dermatitis, dermatitis herpetiformis, insect bites, and drug eruptions [5]. ZSH is likely to be missed or misdiagnosed, and patients may not receive timely antiviral treatment. This causes continuous activation of the VZV, which may induce long lasing herpetic neuralgia, and even postherpetic neuralgia (PHN) due to viral damage to the affected nerves [6].

In rare cases, VZV reactivation manifests as meningitis or encephalitis, in which headache, nuchal rigidity, and/or altered mental status are the predominant symptoms. Such a diagnosis is challenging in the absence of a characteristic rash, as in the case of ZSH. Identifying ZSH as the cause of neurological symptoms becomes urgent in these cases given its communicable nature and the increased risk of morbidity related to CNS infections.

Currently, ZSH can be tested via polymerase chain reaction (PCR) for VZV DNA, analysis of cerebrospinal fluid (CSF) or peripheral blood mononuclear cells for VZV DNA, and anti-VZV IgM and/or IgG antibodies [7]. In the acute setting, however, immunoglobulins are detected only 60% of the time [8]. Treatment often needs to be started empirically when clinical suspicion for varicella is high. In this case series, we describe two cases of ZSH in immunocompetent hosts focusing on their unique presentations, diagnostic considerations, and disease courses.

### Case 1

A 58-year-old female presented to the emergency department with central and right-sided chest pain under her breast. It started a month ago as a dull ache but rapidly increased in severity over the last two days. The pain was described as 9/10 in intensity, and was sharp, burning, and radiating to her back. There was associated shortness of breath. She denied fevers, weight loss, muscle weakness, or loss of sensation. A few days ago, the patient visited her primary care physician who prescribed cyclobenzaprine and recommended physical therapy which provided minimal relief. Her past medical history was significant for type II diabetes mellitus, hypertension, morbid obesity, and hyperlipidemia. Family history and social history were unremarkable with no history of drug, alcohol, or tobacco use.

**Fig. 1:**
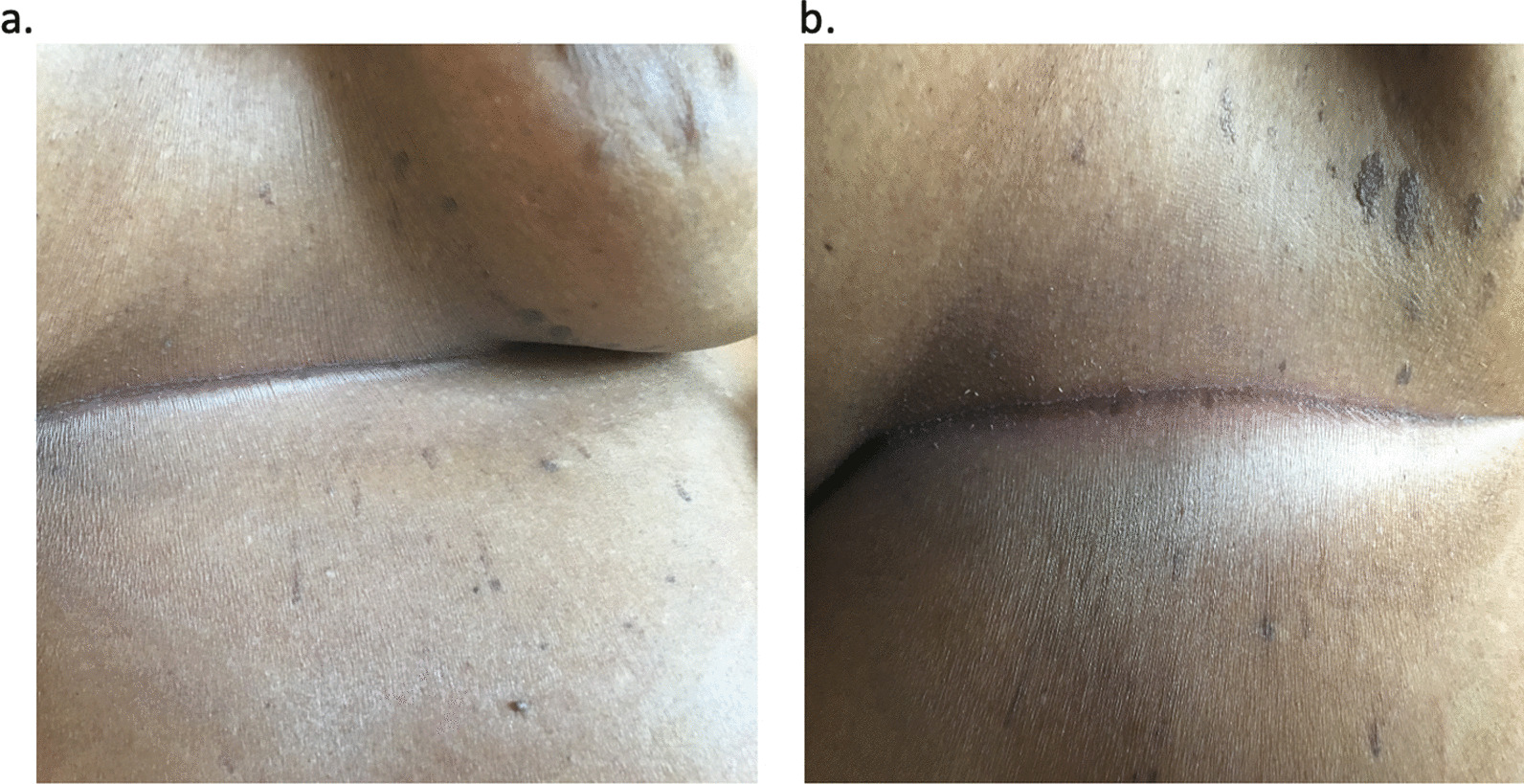
Photograph showing **a** anterior view, and **b** lateral view, of the area under patient 1’s right breast. These painful areas show no rash

On arrival, her vital signs were within normal limits. She appeared in acute distress, but exams of cardiac, pulmonary, and other systems were unremarkable. Dermatologic examination revealed no rash or erythema in the affected region (Fig. 1a, b). Interestingly, we noticed that the pain appeared to localize in the T4-T5 dermatome.

Various causes of chest pain were considered and ruled out. Serial electrocardiograms (EKGs) and cardiac enzymes × 2 followed by a complete cardiac work-up were unremarkable. Chest computed tomography (CT) showed no acute pulmonary pathology. A thoracic spine magnetic resonance imaging (MRI) was only remarkable for mild degenerative changes, ruling out radicular pain. Urine analysis, urine culture, and blood cultures ruled out acute pyelonephritis and the possibility of sepsis.

Once cardiac, pulmonary, and musculoskeletal etiologies were ruled out, the possibility of shingles was considered. However, the absence of any cutaneous manifestations was baffling. Viral serologies for VZV immunoglobulins were sent and the patient was started on oral famciclovir (500 mg, TID), and oral pregabalin (150 mg, q12h). Over the next 48 h, the patient reported significant improvement in her pain. Serologies came back positive for both IgM and IgG. Additionally, patient confirmed a history of childhood varicella infection. A diagnosis of Zoster Sine Herpete was thus made based on positive serologies, T4-T5 dermatomal neuralgia, atypical absence of rash, and robust clinical response to famciclovir and pregabalin.

### Case 2

A 43-year-old female with a past medical history of anxiety, childhood chickenpox, and 18 months post-partum presented to the emergency department with a 2-day history of severe headache and progressive photophobia. Six days prior to presentation, she had an acute-onset sharp pain in the right mid-abdomen radiating to the right flank and mid-back, which had self-resolved. The headache was localized frontally, non-pulsatile, and was associated with nausea, vomiting, and photophobia. She took naproxen without improvement. She denied recent trauma, viral illness, or contact with shingles or chickenpox. VZV IgM was obtained with her primary care physician 2 days ago and was negative.

**Fig. 2:**
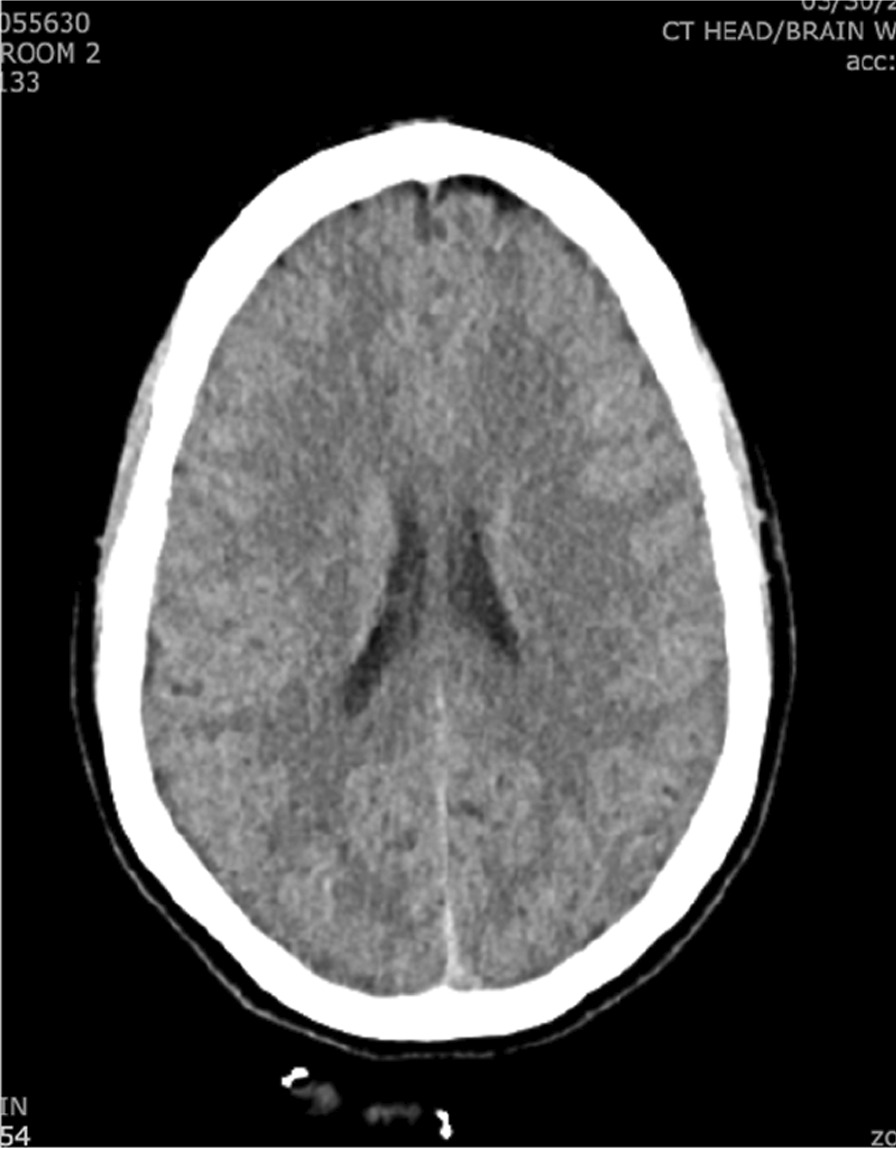
CT head without contrast showed no evidence of intracranial process including masses, bleeding, or elevated intracranial pressure

Vital signs and labs were all within normal limits. Physical exam revealed an uncomfortable-appearing woman but was otherwise unremarkable. Initial skin examination was unrevealing. Basic infectious workups including COVID-19, influenza A/B, and RSV were negative. CT (Fig. 2) and CT Angiography of the head were unremarkable for acute intracranial processes. Lumbar puncture (LP) demonstrated elevated protein (71 mg/dL), normal glucose (59 mg/dL), and markedly elevated mononuclear number with a lymphocytic predominance (95%) in the CSF (Table 1). CSF PCR studies for herpes-simplex virus-1 & 2 (HSV1&2) were sent and were negative. HIV was negative.

**Table 1:** Results of patient 2’s serum Varicella Zoster-IgM and IgG results

Blood test (serum)	Titer	Reference values
Varicella Zoster-IgM	< = 0.90	< or = 0.90 Negative 0.91—1.09 Equivocal > or = 1.10 Positive
Varicella Zoster-IgG	Antibodies present	Antibody present if vaccinated or previous infection

**Fig. 3:**
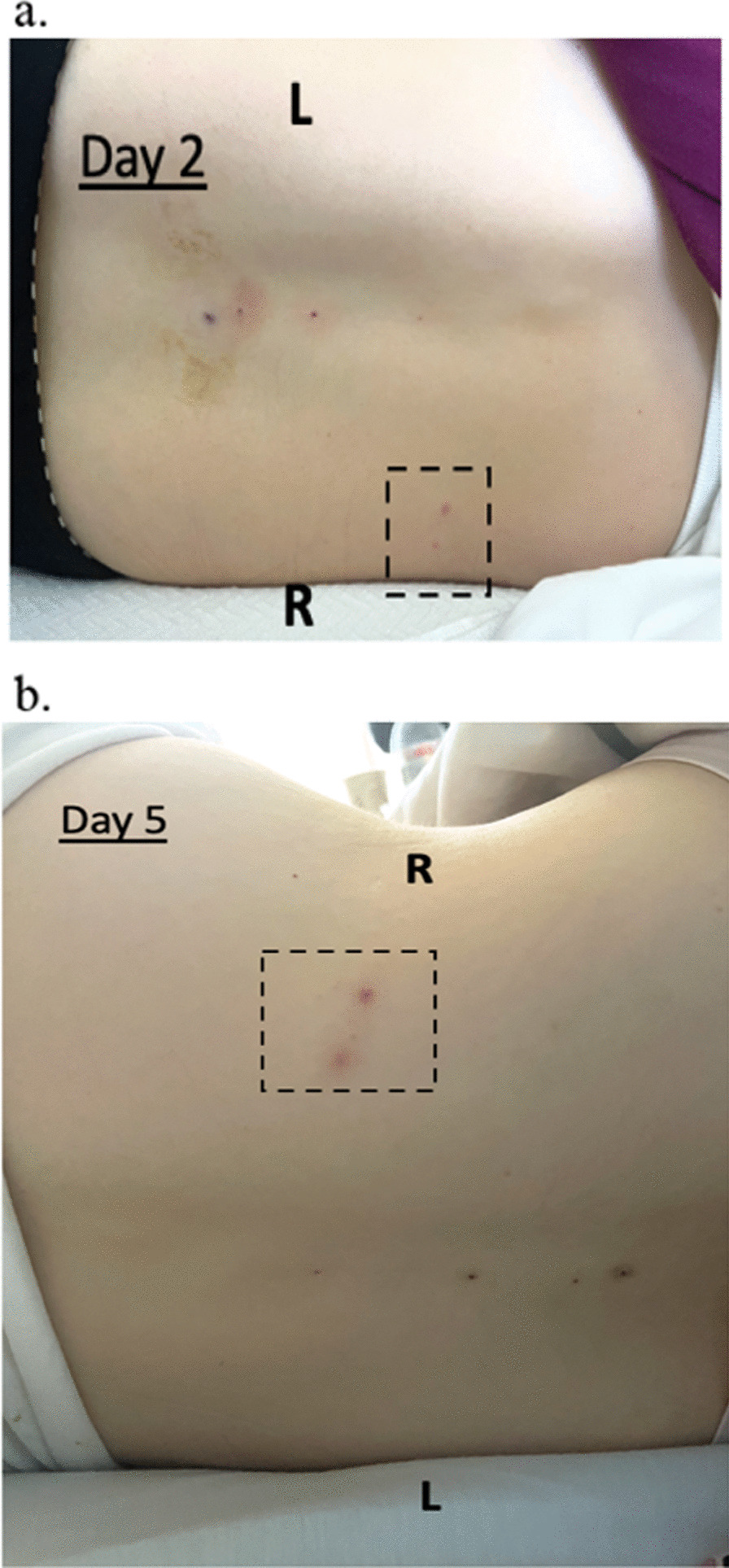
Appearance of patient 2’s right mid-back lesions on hospital **a** day 2; and **b** day 5. Lesions did not change significantly over the course of her hospital stay and treat with intravenous acyclovir. R: patient’s right side; L: patient’s left side

Because her symptoms and CSF results suggested meningitis, empiric intravenous acyclovir (10 mg/kg, TID) and ceftriaxone (2 g, BID) were started. On hospital day 2, revisiting her presenting symptoms revealed a possibility of VZV reactivation. VZV PCR on CSF and repeat VZV serologies were obtained. Results demonstrated negative VZV IgM but positive IgG (Table 1), and positive VZV DNA in CSF, supporting the diagnosis of VZV meningitis. Ceftriaxone was discontinued. Another skin examination at this time (hospital day 2) revealed 2 small lesions at the right posterior flank following T7-T8 dermatomal distribution. Both lesions were slightly raised and erythematous but had no central vesicular changes (Fig. 3a). Over the next several days, her headache gradually resolved on intravenous acyclovir, analgesics, and hydration, and her back lesions remained grossly unchanged (Fig. 3b, c). A repeat LP was obtained on treatment day 9 and showed markedly reduced mononuclear number, slightly decreased glucose (42 mg/dL), normalized protein (34 mg/dL) (Table 2), normal opening pressure (11 cm H_2_O), and undetectable VZV DNA by PCR, confirming robust acyclovir treatment response. We stopped intravenous acyclovir on day 10 of treatment due to the patient’s need to breastfeed, and she was discharged home without complications.

**Table 2:** Results of patient 2’s lumbar puncture before and after a 9-day treatment course with intravenous acyclovir showed markedly decreased CSF protein and mononuclear cell absolute number

CSF findings	Pre-treatment		Post-treatment	
	Tube 1	Tube 2	Tube 1	Tube 2
Appearance	Clear	Clear	Clear	Clear
Color	No color	No color	No color	No color
Glucose (mg/dl) *[ref 50–75]*	59		42	
Protein (mg/dl) *[ref 15–45]*	71		34	
Neutrophils absolute # (/mm^3^)	0	0	0	0
Mononuclear absolute # (/mm^3^) *[ref 0 -5]*	835	670	163	213
Lymphocyte %	95	95	94	87
VZV Qualitative NAT PCR	DNA Detected		No DNA Detected	
HSV 1 Qualitative NAT	No DNA Detected			
HSV 2 Qualitative NAT	No DNA Detected			

## Discussion

Zoster Sine Herpete is a condition manifesting as dermatomal neuralgia in the absence of a rash [1]. It has been proposed to result from either lack of viral migration into the cutaneous tissue after reactivation in the DRG [9] or activation of VZV in enteric ganglia whose axons do not project toward the skin [6]. In both cases, there is associated viral spilling into the CSF which can result in meningitis or encephalitis.

Currently, no studies have investigated the epidemiology of ZSH. However, since the annual rate of HZ ranges from 0.3 to 0.4% in the United States [10], and more than 95% of young adults have positive VZV IgG serologies [11] and are therefore at risk, the actual incidence may be underrecognized due to a lack of clinical suspicion and non-routine testing. In Case 1, a neurological etiology was introduced only after extensive cardiac and musculoskeletal investigations. In retrospect, the burning and radiating nature of her pain warranted the consideration of VZV in the initial workup. Similarly, in Case 2, the patient’s lack of rash on the initial exam made us suspect HSV meningitis before including VZV in the differential diagnosis. High clinical suspicion is warranted for timely work-up and treatment of VZV even when patients present without rash.

In the two cases, VZV immunoglobulins had varied utility in diagnosis and treatment. Patient 1’s positive VZV IgM, IgG, and overall clinical presentation led to the diagnosis of ZSH, whereas patient 2’s negative IgM resulted in a short delay of diagnostic suspicion. The detection of VZV-specific immunoglobulins via enzyme-linked immunosorbent assay has both low sensitivity and low specificity [12], and a positive IgM result cannot differentiate between VZV primary infection, re-infection, and reactivation. In atypical presentations such as ZSH, PCR to detect VZV DNA is a better first-line test because it is rapid, sensitive, and widely available [13]. Additionally, a recent study showed that serum VZV DNA was detected in 69% of patients with ZSH compared to 12.5% in HZ (*P* = 0.0007) at one month after symptom onset [3], validating the utility of PCR even when patients do not present immediately after the pain starts. These results have the potential to change the way ZSH is diagnosed.

Post-herpetic neuralgia is a common complication of shingles and can be a sequela of ZSH as well. Our patient in Case 1 suffered from dermatomal neuralgia for more than a month, whereas our second patient only had transient neuralgia, resolved before presentation to the hospital. Interestingly, a recent study found that the severity of pain and the prevalence of post-herpetic neuralgia were increased in ZSH compared to Herpes Zoster (*P* = 0.0012), leading to more opioid usage by patients with ZSH vs HZ (*P* = 0.0449; OR, 9.00) [3]. Post-herpetic neuralgia has also been found to be more common in immunocompromised hosts [14, 15]. Since Patient 1’s past medical history of diabetes conferred an immunocompromised state, it was reasonable to expect that compared to patient 2, patient 1 was at higher risk for developing persistent pain.

The prevalence of VZV-associated meningitis has been reported to be 8% in a study of 144 patients with aseptic meningitis [16]. A form of disseminated varicella, it tends to occur in immunocompromised hosts. Only a few cases of VZV meningitis following shingles have been reported in immunocompetent individuals [17–19]. Our patient in Case 2 is unique in that she is an immunocompetent, young woman who presented without fever or a classic rash. At presentation, her dermatomal neuralgia had resolved, and she had no classic lesions, both adding to the diagnostic complexity. Additionally, her lesions’ appearance did not change during the treatment course, rendering it uncertain whether they did represent cutaneous migration of VZV. We, therefore, categorized this case as ZSH meningitis, an even rarer form of varicella meningitis. To date, there have only been several reported cases of varicella meningitis associated with no rash [9, 20, 21] or late-onset rash [22], although the incidence may be underestimated due to non-routine testing. Due to the potentially devastating sequelae of CNS infections, we advocate keeping VZV in the differential diagnoses of meningitis, and strongly echo Kangath et al.’s proposal [18] to include VZV PCR as part of routine CSF testing following LP for presumed meningitis.

## Conclusions

Zoster Sine Herpete (ZSH) is a rare presentation of VZV reactivation. It can be seen in both immunocompromised and immunocompetent individuals, and can be associated with post-herpetic neuralgia, especially in immunocompromised hosts. ZSH is likely underreported and underdiagnosed due to the atypical absence of a rash and non-routine testing. The clinicians should play a role in educating patients and have a low threshold of conducting thorough workup, especially in immunocompromised patients presenting with herpes zoster-like symptoms without rash, to diagnose and treat such conditions in a timely manner. Early diagnosis with VZV PCR and treatment with appropriate antivirals can significantly improve patients’ pain and prevent life-threatening neurological sequelae such as meningitis. These two cases highlight the importance of including ZSH as a differential diagnosis in relevant clinical scenarios and call for astute clinical judgement and a high index of suspicion to prevent potential significant complications of ZSH.

## Data Availability

Data sharing not applicable to this article as no datasets were generated or analyzed during the current study.

## Abbreviations

VZV: Varicella-zoster virus
ZSH: Zoster Sine Herpete
IgG/IgM: Immunoglobulin G/M
DNA: Deoxyribonucleic acid
HZ: Herpes Zoster
DRG: Dorsal root ganglia
CNS: Central nervous system
PCR: Polymerase chain reaction
CSF: Cerebrospinal fluid
EKG: Electrocardiogram
CT: Computed tomography
MRI: Magnetic resonance imaging
TID: Three times a day
COVID-19: Coronavirus disease of 2019
RSV: Respiratory syncytial virus
LP: Lumbar puncture
HSV: Herpes simplex virus
BID: Twice a day
